# Chyloma: A Manifestation of Chyle Leak Six Months After Neck Dissection

**DOI:** 10.7759/cureus.20476

**Published:** 2021-12-17

**Authors:** Patricia Li-Min Tay, Siu Cheng Loke, Teresa Hui Xian Ng, Ming Yann Lim, Hao Li

**Affiliations:** 1 Otorhinolaryngology, Tan Tock Seng Hospital, Singapore, SGP; 2 Diagnostic Radiology, Tan Tock Seng Hospital, Singapore, SGP; 3 Nutrition & Dietetics, Tan Tock Seng Hospital, Singapore, SGP

**Keywords:** chylous leakage, neck dissection, tisseel, tongue carcinoma, streptococcus agalactiae, chyle leak, chyloma, chylocele, thoracic duct, fat-free diet

## Abstract

Chyle leaks after a neck dissection usually manifest within the immediate postoperative period. However, masked chyle leaks may present as a chyloma months later.

A 54-year-old male patient with squamous cell carcinoma of the tongue underwent bilateral neck dissection, subtotal glossectomy, anterolateral thigh flap reconstruction and postoperative radiotherapy. Intraoperatively, chyle leak was encountered in level IV of the left neck. We managed it by ligation of the thoracic duct, application of Tisseel™ sealant (Baxter Inc., Illinois, USA) and one week of prophylactic fat-free feeds. Six months later, an asymptomatic chyloma of the left neck was identified on surveillance MRI. Five weeks after the diagnosis, streptococcal infection developed within the chyloma. However, initiation of fat-free diet, serial aspiration, pressure dressing and antibiotic therapy allowed the chyloma to resolve within two weeks. Further surveillance MRI over three years showed no recurrence of the chyloma.

Low-volume chyle leaks may manifest as an occult chyloma. Prophylactic measures cannot replace meticulous ligation of chylous channels in left level IV neck dissection.

## Introduction

Chyle leak post neck dissection is a rare but serious complication that poses a challenge to the head and neck surgeon. Its incidence ranges between 1-5% in neck dissections [1] and more commonly occurs with dissection of the left side in the proximity of the thoracic duct [1-3]. Chyle leaks are often detected within a week of surgery, typically upon resumption of oral diet. However, we report a case of an occult chyle leak presenting as an asymptomatic chyloma detected on magnetic resonance imaging six months after neck dissection.

## Case presentation

The patient is a 54-year-old male diagnosed with a clinically T4aN2cM0 (American Joint Committee on Cancer 8th Edition Staging Manual) squamous cell carcinoma arising from the right side of the oral tongue. He underwent subtotal glossectomy, bilateral modified radical neck dissection, free anterolateral thigh flap reconstruction and postoperative radiotherapy. During this patient’s left level IV neck dissection, chyle leak was encountered and controlled with ligation of the thoracic duct followed by the application of Tisseel™ sealant (Baxter Inc., Illinois, USA). We placed closed suction drains in his neck and prescribed fat-free feeds (Resource™ fruit beverage, Nestlé Inc., Vevey, Switzerland) exclusively in the first postoperative week. In the second week, we re-introduced fat into his feeds and did not observe neck erythema, swelling or milky drain output that indicated a chyle leak. The suction drains were removed on postoperative day 9 when the output decreased to less than 20 millilitres per day.

**Figure 1:**
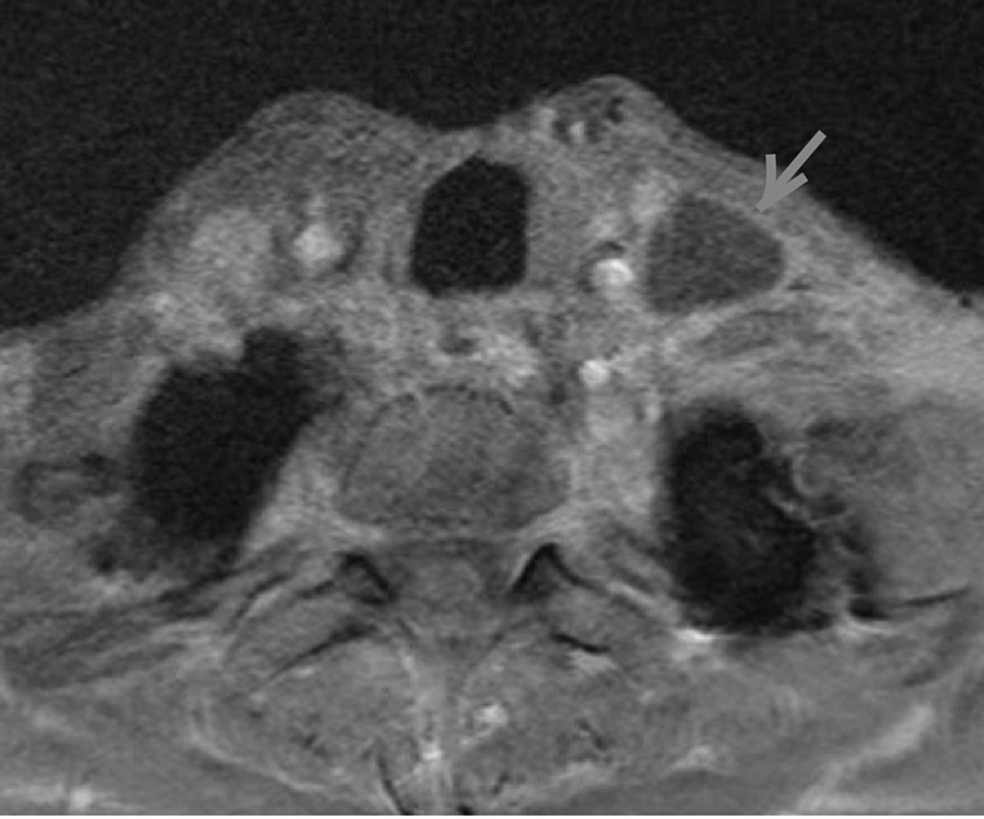
Arrow points to the chyloma on axial T1-weighted, gadolinium-enhanced MRI of the neck. The chyloma was located posterolateral to the left internal jugular vein at the level of the trachea (left level IV of the neck). At this location, the thoracic duct is expected to traverse the neck before it drains into the confluence of the internal jugular and subclavian veins. The chyloma was a 3.4 x 2.2 x 1.7cm circumscribed cystic lesion that was hypointense on T1 and did not enhance with the administration of gadolinium. The rest of the neck was unremarkable.

**Figure 2:**
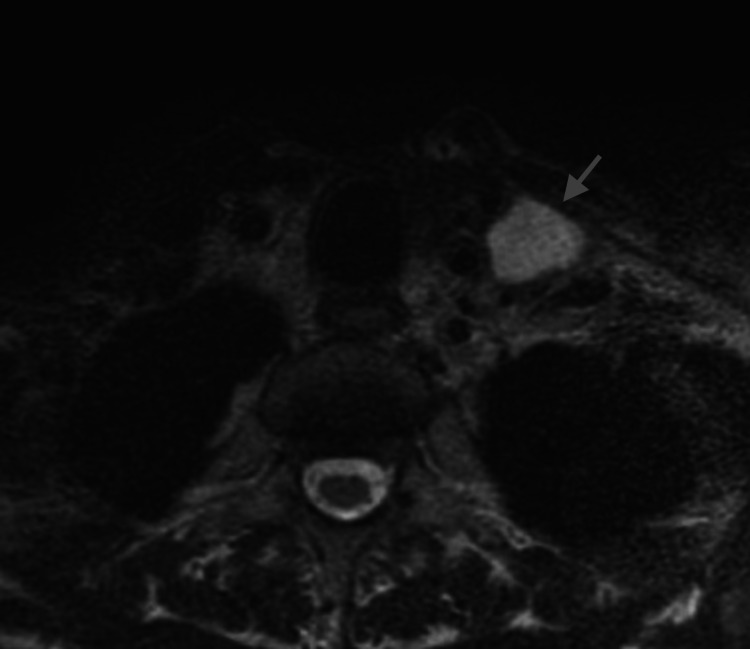
Arrow points to the chyloma on axial T2-weighted, non-enhanced MRI of the neck. The chyloma was hyperintense on T2.

**Figure 3:**
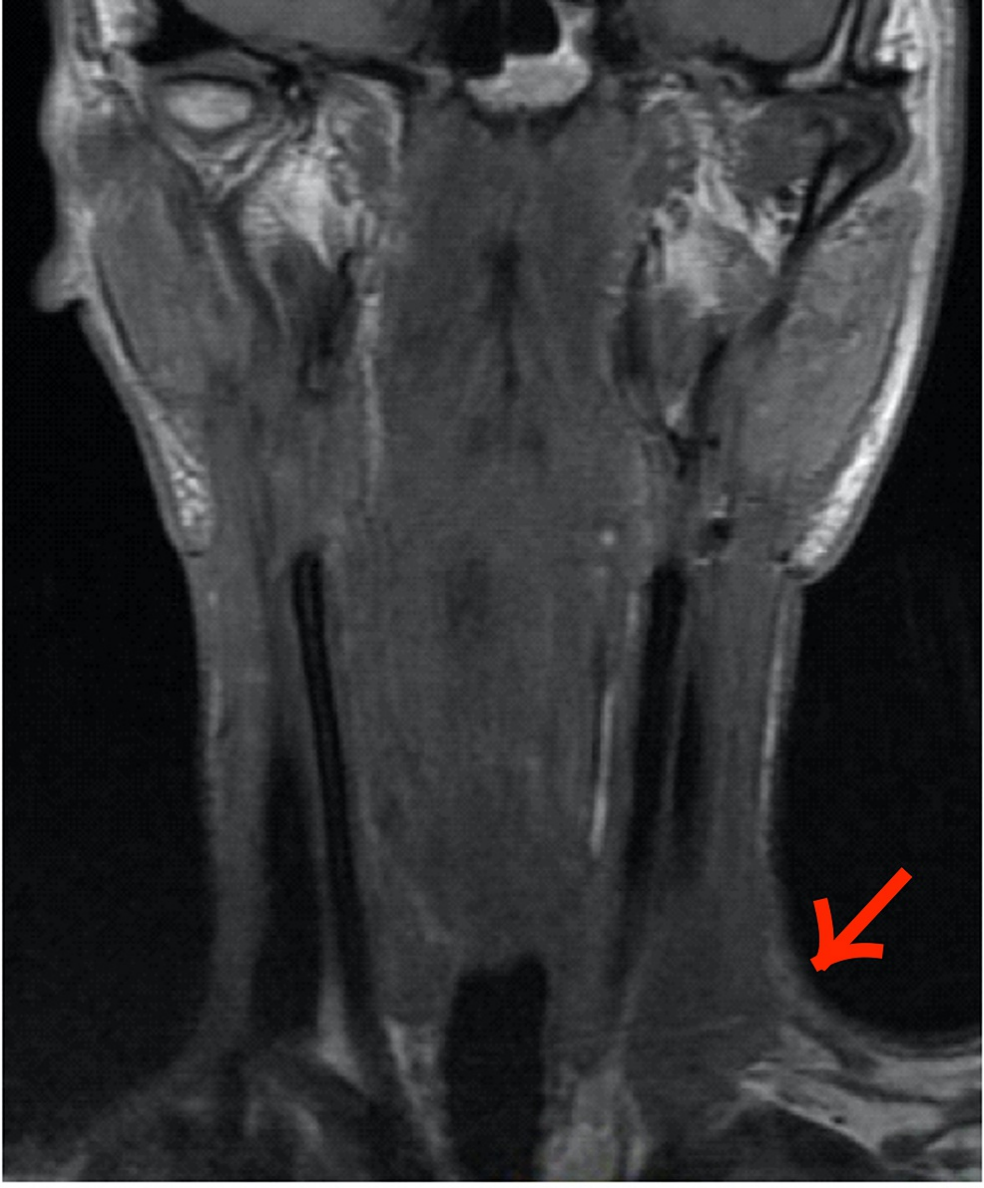
Arrow points to the chyloma on coronal T1-weighted, non-enhanced MRI of the neck. Together with Figure 1, this coronal view of the chyloma shows its location in left level IV of the neck where the thoracic duct is expected to be encountered during neck dissection. The chyloma appeared as a circumscribed hypointense lesion on T1.

**Figure 4:**
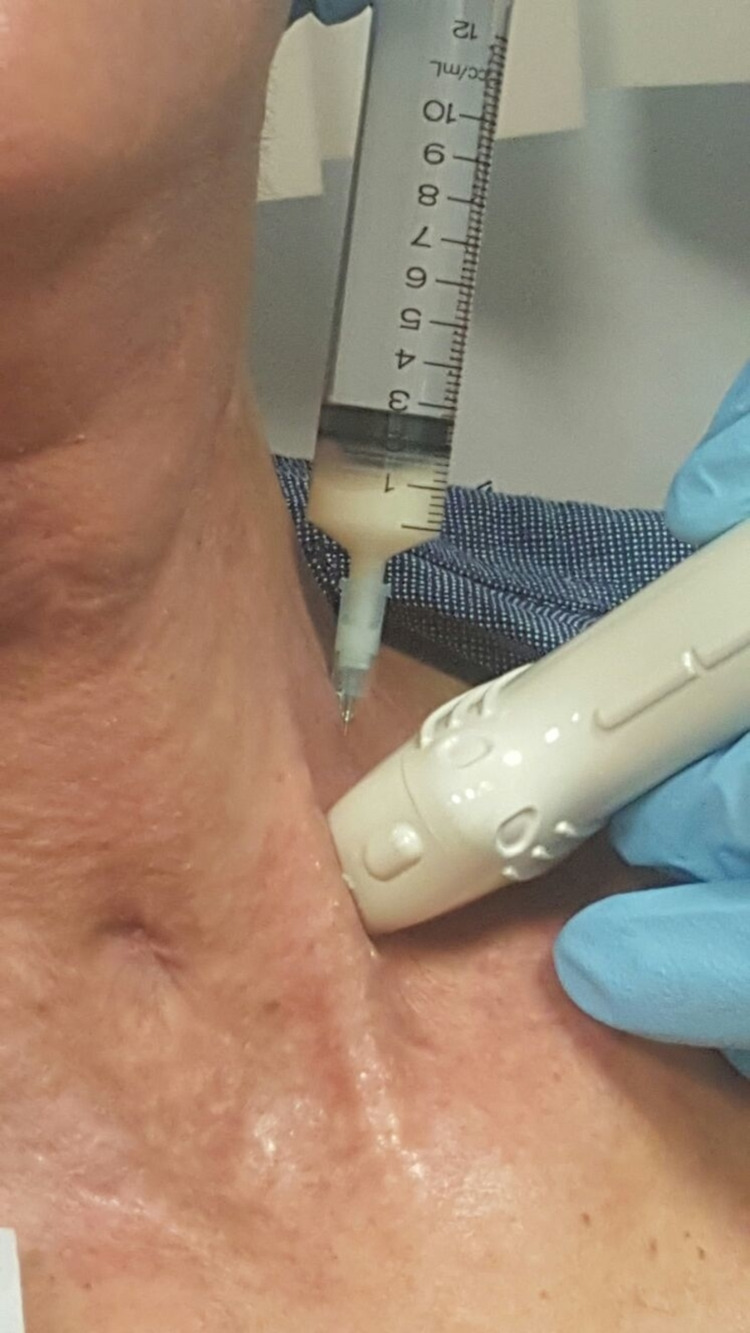
Milky aspirate revealing the diagnosis of chyloma.

Six months after the surgery and three months after the completion of radiotherapy, an asymptomatic fluid collection in level IV of the left neck was identified on routine magnetic resonance imaging (MRI) performed to assess the presence of residual cancer. This fluid collection appeared as a 3.4 centimetre circumscribed, T1- hypointense, T2-hyperintense, non-enhancing lesion posterolateral to the internal jugular vein (Figures 1-3). Neck examination revealed no swelling, erythema, warmth or tenderness. Ultrasound-guided fine-needle aspiration yielded a milky aspirate revealing the clinical diagnosis of a chyloma (Figure 4). The patient was immediately commenced on Resource™ fruit beverage. Upon reviewed a week later, he remained asymptomatic and did not manifest erythema, swelling or tenderness of his neck. He was instructed to gradually re-introduce fat into his diet. However, five weeks later, he developed tenderness, erythema and swelling over level IV of the left neck suggestive of infection of the chyloma. Needle aspiration again revealed milky fluid and the bacteriology of the aspirate grew Streptococcus agalactiae. We started him on oral co-amoxiclav, applied a pressure dressing to the left level IV of his neck, increased the frequency of aspiration to every other day and commenced a fat-free diet again. A week later, the aspirate from the left neck turned haemoserous. By the 10th day, the volume of the aspirate decreased from 5 to 1 millilitre. On the 14th day, we progressed the patient to a low-fat diet and on the 28th day, a regular diet. MRI of his neck done for the surveillance of cancer recurrence 4.5 months later revealed resolution of this chyloma and regular MRI over the next three years showed no recurrence of either the chyloma or the squamous cell carcinoma.

## Discussion

Chylomas, otherwise known as chyloceles or lymphoceles if a cyst wall can be identified, have been occasionally reported as a delayed manifestation of chyle leak after neck surgery [4-6]. Besides the neck, chylomas can also occur along the more proximal course of the thoracic duct from the cisterna chyli to the mediastinum [7,8]. Large chylomas can cause swellings or compressive symptoms although small ones can be occult [4-8]. Management may be expectant in asymptomatic patients, but proactive drainage [5], sclerotherapy [9], surgical exploration [6] in conjunction with a fat-free diet [10] can be performed as definitive treatment. The use of somatostatin analogue [1] and parenteral nutrition as an adjunct is also not unreasonable in cases of refractory chyle leak [11]. It is unclear whether asymptomatic chylomas should be proactively treated. However, without adequate drainage, our patient developed a bacterial infection within the chyloma, although one may argue that the needle aspiration could have triggered the infection as well. Nonetheless, we demonstrate that a small chyloma, even though infected, can resolve within days after serial aspiration, pressure dressing, antibiotics and a fat-free diet. Moreover, it intrigues us why chylomas may occur without signs of chyle leak during the initial postoperative period. Perhaps, a contained low-volume chyle leak arising from a branch of the thoracic duct injured intraoperatively was the cause of chyloma in this patient. In addition, the fat-free feeds prescribed to the patient postoperatively could have made chyle serous in appearance, thus masking the detection of chyle leak by inspection of drain fluid. Prophylactic measures such as fibrin sealant and fat-free diet cannot replace gentle handling and thorough ligation of chylous channels in level IV neck dissection. Prevention is more important than cure in chyle leaks.

## Conclusions

Chyloma can be a manifestation of occult chyle leak after neck surgery. It can be successfully treated with the same principles of managing overt chyle leaks. In the prevention of chyle leaks, Tisseel™ sealant or fat-free feeds cannot replace meticulous ligation of chyle ducts in left level IV neck dissection. The natural history of chylomas should also be studied in order to help us understand whether treatment is indeed necessary in asymptomatic patients.
